# A longitudinal study of anti-SARS-CoV-2 antibody seroprevalence in a random sample of the general population in Hiroshima in 2020

**DOI:** 10.1265/ehpm.22-00016

**Published:** 2022-07-07

**Authors:** Aya Sugiyama, Fumie Okada, Kanon Abe, Hirohito Imada, Serge Ouoba, Bunthen E, Md Razeen Ashraf Hussain, Masayuki Ohisa, Ko Ko, Shintaro Nagashima, Tomoyuki Akita, Shinichi Yamazaki, Michiya Yokozaki, Eisaku Kishita, Junko Tanaka

**Affiliations:** 1 Department of Epidemiology, Infectious Disease Control and Prevention, Graduate School of Biomedical and Health Sciences, Hiroshima University, Hiroshima, Japan; 2 Hiroshima Prefecture Health and Welfare Bureau, Hiroshima, Japan; 3 Unité de Recherche Clinique de Nanoro (URCN), Institut de Recherche en Science de la Santé (IRSS), Nanoro, Burkina Faso; 4 Payment Certification Agency, Ministry of Health, Cambodia; 5 Division of Clinical Laboratory Medicine, Hiroshima University Hospital, Hiroshima, Japan

**Keywords:** SARS-CoV-2, Antibody test, Prevalence, Seroconversion, General population, Random sampling, Japan

## Abstract

**Background:**

This longitudinal study aimed to determine chronological changes in the seroprevalence of prior SARS-CoV-2 infection, including asymptomatic infections in Hiroshima Prefecture, Japan.

**Methods:**

A stratified random sample of 7,500 residents from five cities of Hiroshima Prefecture was selected to participate in a three-round survey from late 2020 to early 2021, before the introduction of the COVID-19 vaccine. The seroprevalence of anti–SARS-CoV-2 antibodies was calculated if at least two of four commercially available immunoassays were positive. Then, the ratio between seroprevalence and the prevalence of confirmed COVID-19 cases in Hiroshima was calculated and compared to the results from other prefectures where the Ministry of Health, Labour and Welfare conducted a survey by using the same reagents at almost the same period.

**Results:**

The numbers of participants in the first, second, and third rounds of the survey were 3025, 2396, and 2351, respectively and their anti-SARS-CoV-2 antibodies seroprevalences were 0.03% (95% confidence interval: 0.00–0.10%), 0.08% (0.00–0.20%), and 0.30% (0.08–0.52%), respectively. The ratio between the seroprevalence and the prevalence of confirmed COVID-19 cases in Hiroshima was 1.2, which was smaller than that in similar studies in other prefectures.

**Conclusions:**

The seroprevalence of anti–SARS-CoV-2 antibodies in Hiroshima increased tenfold in a half year. The difference between seroprevalence and the prevalence of confirmed COVID-19 cases in Hiroshima was smaller than that in other prefectures, suggesting that asymptomatic patients were more actively detected in Hiroshima.

**Supplementary information:**

The online version contains supplementary material available at https://doi.org/10.1265/ehpm.22-00016.

## Background

An outbreak of pneumonia caused by the severe acute respiratory syndrome coronavirus 2 (SARS-CoV-2) virus was first reported to have occurred in Wuhan City, Hubei Province, China, in December 2019 [1–3]. Thereafter, it spread across the world in several weeks [4–6], and the World Health Organization (WHO) designated the outbreak as a pandemic on March 11, 2020. As of September 13, 2020, the cumulative number of SARS-CoV-2 infections in Japan was reported to be 1.64 million [7].

Coronavirus disease 19 (COVID-19), caused by the SARS-CoV-2 virus, may result in severe symptoms in high-risk populations such as older individuals or those with underlying diseases. On the other hand, some patients are asymptomatic but can still transmit the virus to others, making it difficult to contain its spread [8–16]. There is a gap between the actual and reported numbers of people with prior infection [17–20]. Infection control measures can be developed based on epidemiological data regarding the number of people who have experienced symptomatic or asymptomatic SARS-CoV-2 infection and who have protective antibodies against SARS-CoV-2 [18, 21, 22]. The status of the COVID-19 outbreak and the availability of data on the number of infected people vary by country and region, and therefore the spread of the disease needs to be monitored in different areas. Hiroshima Prefecture consists of 23 municipalities with a population of 2.79 million. At the time of writing this manuscript, September 13, 2021, the officially reported cumulative number of SARS-CoV-2 infections in Hiroshima Prefecture was approximately 20,000 [23]. In 2020, before COVID-19 vaccines were introduced, we conducted a three-round survey on the seroprevalence of anti–SARS-CoV-2 antibodies in a randomly selected general population in Hiroshima Prefecture.

This study aimed to determine the prevalence of prior SARS-CoV-2 infection and its changes over time, and to compare the gap between the seroprevalence and the prevalence of confirmed COVID-19 cases with that in other prefectures. The present study was supported by the Hiroshima Prefectural Government and conducted as part of the “Government-Academia Collaborative Project between Hiroshima Prefecture and Hiroshima University to Develop a Testing and Research System for COVID-19.”

## Methods

A large-scale epidemiological three-round survey on the seroprevalence of anti–SARS-CoV-2 antibodies was conducted in a general adult population in Hiroshima Prefecture of Japan. Individuals were selected based on the stratified random sampling technique using the Basic Resident Register.

### Target cities

**Fig. 1:**
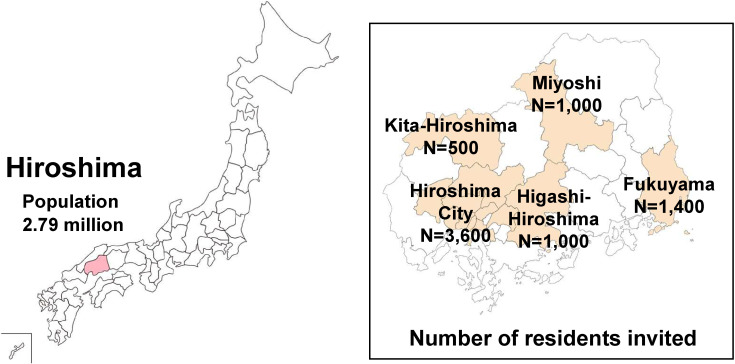
Study setting in Hiroshima prefecture and number of residents invited to participate Of the 23 cities in Hiroshima prefecture, five cities were selected for the study. The number of participants invited in each city was adjusted to the total population in the city.

The study was conducted in five of the 23 cities of Hiroshima Prefecture: Hiroshima, Fukuyama, Higashi-Hiroshima, Miyoshi, and Kita-Hiroshima. Figure 1 presents the study area with the selected cities and the number of residents invited to participate. Hiroshima and Fukuyama are urban areas with populations of 1,196,138 and 469,960, respectively, while Higashi-Hiroshima, Miyoshi and Kita-Hiroshima are rural areas with populations of 187,718, 53,556 and 18,780, respectively.

### Sample size

The sample size necessary to estimate the seroconversion rate was calculated to be 5,680 using the following formula:

$$n=\frac{1.96^{2}p(1-p)}{d^{2}}\cdot \frac{1}{R}$$

(p, expected seroconversion rate 0.20%; d, absolute accuracy 0.15%; R, participation rate 60%). Taking the population ratios of the five cities into account, the overall sample size was determined to be 7,500 (3,600 in Hiroshima, 1,400 in Fukuyama, 1,000 in Higashi-Hiroshima, 1,000 in Miyoshi, and 500 in Kita-Hiroshima) (Table 1).

**Table 1:** Sampling procedure in 5 cities of Hiroshima prefecture

**City**	**Population**	**Proportion of population in each city**	Required sample size*	**Number of residents invited**	**Proportion of population invited**
**Total (5 cities in ** **Hiroshima prefecture)**	**1,925,152**	**100%**	**5,680**	**7,500**	**0.39%**
Hiroshima	1,196,138	62.1%	3,527	3,600	0.30%
Fukuyama	469,960	24.4%	1,386	1,400	0.30%
Higashi-Hiroshima	187,718	9.8%	557	1,000	0.53%
Miyoshi	53,556	2.7%	153	1,000	1.90%
Kita-Hiroshima	18,780	1.0%	57	500	2.66%

*Required total sample size was calculated assuming an antibody positivity rate of 0.20%, an absolute accuracy of 0.15%, and a participation rate of 60%. The required sample size for each city was determined by applying the required total sample size to the proportion of the population in each city.

### Participant sampling method

The residents in each region were stratified by sex and age (20–39 years; 40–59 years; 60–84 years). Study participants were then selected based on the stratified random sampling technique using the Basic Resident Register of each city.

### Survey method

The survey was conducted in three rounds (August to September 2020, October to November 2020, and January to February 2021) in the same population. In each round, an invitation letter was mailed to the candidate study participants to request their participation in the survey. Those who provided written consent completed a self-administered questionnaire and underwent collection of 10 mL of blood at the study site. The questionnaire items addressed basic characteristics such as age group and sex, subjective symptoms within the past 6 months, personal infection prevention measures, history of SARS-CoV-2 infection, history of contact with individuals with SARS-CoV-2 infection, behavioral history, medical history, and smoking/alcohol use history.

### Laboratory assays

To ensure the accuracy of the test results, we performed antibody testing using four different immunoassays: Vitros Anti–SARS-CoV-2 Total (Ortho Clinical Diagnostics, Rochester, NY, United States; target, S protein; for chemiluminescent enzyme immunoassay, sensitivity: 98.5%, specificity 100% [24]), Vitros Anti–SARS-CoV-2 IgG (Ortho Clinical Diagnostics, Rochester, NY, United States; target, S protein; for chemiluminescent enzyme immunoassay, sensitivity: 98.7%, specificity 100% [24]), Elecsys Anti–SARS-CoV-2 (Roche Diagnostics International Ltd, Rotkreuz, Switzerland, target, N protein; for electrochemiluminescent immunoassay, sensitivity: 99.5%, specificity 99.8% [25]), and Architect Abbott SARS-CoV-2 IgG (Abbott Diagnostics, Chicago, USA; target, N protein; for chemiluminescent immunoassay, sensitivity: 100%, specificity 99.9% [26]). All immunoassays were approved by the US Food and Drug Administration (FDA) and were assessed in Japan [27]. Although we used FDA-approved immunoassays, we needed to be cautious about their evaluation as they were newly developed reagents. Furthermore, it should be considered that when testing a population with a significantly low prevalence, false positives are more likely detected because the positive predictive value becomes lower even if the test accuracy is high enough [16, 28–30].

We also assessed the presence of neutralizing antibodies to SARS-CoV-2 in samples positive for at least one of the four reagents, using the GenScript SARS-CoV-2 Surrogate Virus Neutralization Test (sVNT) (GenScript, USA), an ELISA test mimicking the viral neutralizing process. The sVNT was considered positive when the inhibition rate was ≥30%.

### Definition of anti-SARS-CoV-2 antibody positivity

The definition of antibody positivity in this study was determined by comprehensively considering the antibody measurement results using the four immunoassays, the neutralizing antibody detection test, the changes over time in the antibody measurement results, and the subjective symptoms. As a result, a study participant was considered positive for anti–SARS-CoV-2 antibodies when the results of at least two different immunoassays were positive.

### Cumulative number of SARS-CoV-2 infections in Hiroshima Prefecture

The cumulative number of SARS-CoV-2 infections in Hiroshima Prefecture was estimated based on the seroprevalence of anti–SARS-CoV-2 antibodies and the total population of Hiroshima prefecture. The prevalence of SARS-CoV-2 infections, based on the official report number of confirmed COVID-19 cases in Hiroshima Prefecture, was compared with the seroprevalence determined by antibody testing in this study to estimate the number of infections that were not reported.

### Anti–SARS-Cov-2 antibody seroconversion rate

The anti–SARS-Cov-2 antibody seroconversion rate was calculated using the person-year method. A positive test (prior infection) was defined as positive results with at least two different immunoassays. The seroconversion rate was estimated in participants who responded to the survey in at least two of the three rounds and who tested negative for anti–SARS-CoV-2 antibodies at the first round.

### Comparison with the seroprevalence in other prefectures

The test results from the third round of this study and the results of the survey on seroprevalence of anti–SARS-CoV-2 antibodies, which was conducted by the Ministry of Health, Labour, and Welfare (MHLW) of Japan in a randomly sampled general population in Tokyo, Osaka, Miyagi, Aichi, and Fukuoka prefectures in December 2020 [31], were compared with the Japanese government official report of confirmed COVID-19 cases. Both surveys were conducted during the similar period. In the MHLW survey [31], anti-SARS-CoV-2 antibody positivity was defined as positive results with two immunoassays manufactured by Roche Diagnostics International and Abbott Diagnostics, which were the same reagents as our survey. To match the criteria, we used the results of these two immunoassays to compare our findings with those of the MHLW survey.

### Statistical analysis

The positivity rate was estimated with the Wald’s 95% confidence interval (CI), i.e.

$$\hat{p}\pm 1.96\sqrt{\frac{\hat{p}(1-\hat{p})}{n}}$$

where, *n* is the sample size, *X* is the number of positive cases and $\hat{p}=X/n$ .

Incidence rate = *r*/*T* where *r* and *T* are the number of new infections detected during the observational period and the total observed person-months, respectively. 95% confidence intervals (CI) for incidence rates of new infections were based on a Poisson distribution as follows:

$$\text{95\% lower limit of incidence rate}=\chi_{2r}^{2}(0.975)/(2T)$$

$$\text{95\% upper limit of incidence rate}=\chi_{2r+2}^{2}(0.025)/(2T)$$

where $\chi_{2r}^{2}(0.975)$ was the upper 97.5% point of a χ^2^ distribution with 2*r* degrees of freedom (df) and $\chi_{2r+2}^{2}(0.025)$ the upper 2.5% point of a χ^2^ distribution with 2*r* + 2 df. Microsoft Excel was used for the calculation.

## Results

### Study participants

**Fig. 2:**
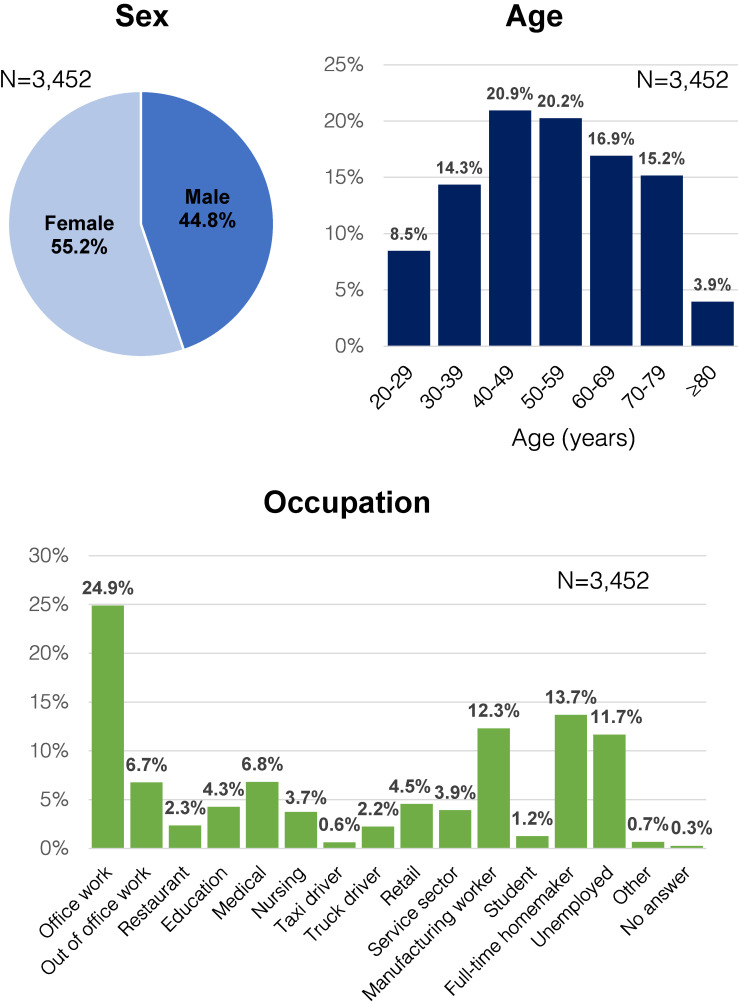
Characteristics of residents who participated in at least one round of the study (N = 3,452) Distribution of sex, age, and occupational characteristics of the residents who participated at least once in all three rounds of this study. Females (55.2%), office workers (24.9%), and residents in their 40s (20.9%) were the most represented.

Among 7,500 candidate participants, 3,025 (40.3%), 2,396 (31.9%), and 2,351 (31.4%) participated in the survey on the first, second, and third rounds, respectively. A total of 1,766 residents participated in all three rounds, while 2,544 participated at least twice and 897 participated only once. The number of participants who responded at least once was 3,452, including 55.2% males and 44.8% females. Those in their 40s comprised the largest age group (20.9%), followed by those in their 50s (20.2%); and their most common job was office workers (24.9%), followed by full-time homemakers (13.7%) and manufacturing workers (12.3%) (Fig. 2).

### Anti–SARS-CoV-2 antibody-positive participants

A total of 72 samples obtained from 39 participants were tested positive for antibodies with at least one of the four reagents. The results of the antibody measurement using the four reagents, the neutralizing antibody detection test, self-reported subjective symptoms, history of COVID-19 infection and of contact with COVID-19 patients by each study round are shown in Supplementary Table 1. Based on these results, we defined the positivity of anti–SARS-CoV-2 antibodies when the results of at least two different immunoassays were positive in this study. The numbers of participants who tested positive for anti–SARS-CoV-2 antibodies with at least two immunoassays on the first, second, and third rounds of the survey were 1, 2, and 7, respectively (Table 2). Excluding positive results from the same participants who participated at least twice, the total number of participants who tested positive was seven. Two participants (Case 1, male in his 70s; Case 7, female in her 80s) reported no past infection, no known close contacts with SARS-CoV-2 infected patients, and no COVID-19 symptoms. Another participant (Case 6, male in his 40s) who reported no past infection and no known close contacts with a patient with COVID-19, reported a history of dysosmia 3 months prior to the survey. Another participant (Case 2, male in his 60s) reported close contact with a SARS-CoV-2–positive patient, had a history of fever within 6 months, but did not report any past infection. The other three participants with positive results (Case 3, female in her 60s; Case 4, male in his 30s; Case 5, male in his 50s) reported that they had COVID-19 symptoms and were diagnosed and received treatment in December 2020 (Supplementary Table 1).

**Table 2:** Result of antibody testing during the three surveys in 5 cities of Hiroshima prefecture

	**Ortho Anti-S Total (IgG, ** IgM, IgA)*	Ortho Anti-S IgG**	Roche anti-N IgG***	Abbott anti-N IgG****	**First round** **(N = 3,025)**	**Second round** **(N = 2,396)**	**Third round** **(N = 2,351)**
**At least two positive tests from two different immunoassays**	Positive (+)	Positive (+)	Positive (+)	Positive (+)	1	1	5
	Positive (+)	Positive (+)	Negative (−)	Positive (+)	0	0	1
	Positive (+)	Positive (+)	Negative (−)	Negative (−)	0	1	1

**Negative tests on all immunoassays or only one immunoassay positive**	Positive (+)	Negative (−)	Negative (−)	Negative (−)	1	4	2
	Negative (−)	Positive (+)	Negative (−)	Negative (−)	7	7	7
	Negative (−)	Negative (−)	Positive (+)	Negative (−)	3	5	8
	Negative (−)	Negative (−)	Negative (−)	Positive (+)	8	4	6
	Negative (−)	Negative (−)	Negative (−)	Negative (−)	3,005	2,374	2,321

Antibody positivity was defined as at least two positive results from the four immunological tests.

*Total antibody (IgG, IgM, IgA) to SARS-CoV-2 Spike protein detected by chemiluminescent enzyme immunoassay (CLEIA) using VITROS Anti-SARS-CoV-2 Total (Ortho Clinical Diagnostics, USA)

**Immunoglobulin G (IgG) to SARS-CoV-2 Spike protein detected by chemiluminescent immunoassay (CLEIA) using VITROS Anti-SARS-CoV-2 IgG (Ortho Clinical Diagnostics, USA)

***IgG to SARS-CoV-2 nucleocapsid protein detected electrochemiluminescence immunoassay (ECLIA) using ELECSYS Anti-SARS-CoV-2 (incl. IgG) (Roche Diagnostics, Swiss)

****IgG to SARS-CoV-2 nucleocapsid protein detected by chemiluminescent immunoassay (CLIA) using ARCHITECT SARS-CoV-2 IgG (Abbott Laboratories, USA)

### Changes in the seroprevalence of anti–SARS-CoV-2 antibodies over time

The seroprevalence of anti–SARS-CoV-2 antibodies on the first, second, and third rounds of the survey were 0.03% (1/3,025; 95%CI, 0.00–0.10%), 0.08% (2/2,396; 95%CI, 0.00–0.20%), and 0.30% (7/2,351; 95%CI, 0.08–0.52%), respectively. Based on these seroprevalence rates, the estimated numbers of people with prior SARS-CoV-2 infection in the population of 2.79 million in Hiroshima Prefecture on the first, second, and third rounds of the survey were 922 (95% CI, 0–2,730), 2,329 (95% CI, 0–5,555), and 8,307 (95% CI, 2,162–14,452), respectively.

### Comparison with the officially reported number of confirmed SARS-CoV-2 cases

**Fig. 3:**
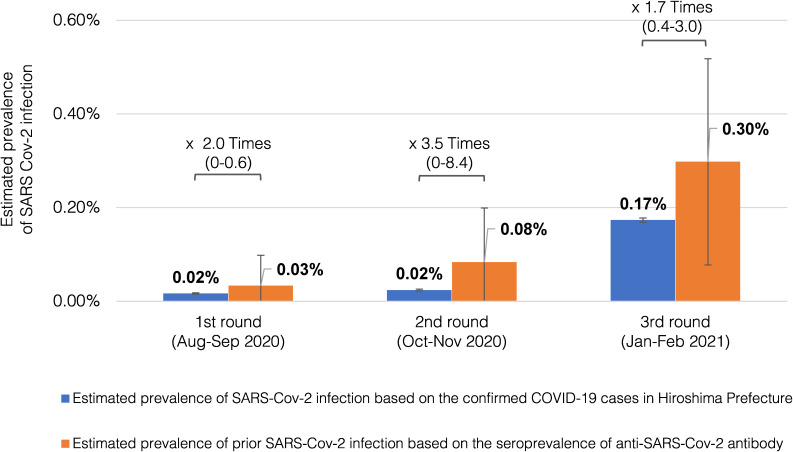
Comparison of anti-SARS-Cov-2 antibody seroprevalence with the prevalence of confirmed COVID-19 cases in Hiroshima Prefecture Blue bars represent the prevalence of SARS-CoV-2 infections in Hiroshima Prefecture, estimated by dividing the number of officially announced COVID-19 cases by the total population in Hiroshima Prefecture. Orange bars show the seroprevalence of anti-SARS-Cov-2 antibody, defined as at least two positive results from the four immunoassays (Vitros Anti–SARS-CoV-2 Total, Vitros Anti–SARS-CoV-2 IgG, Roche Elecsys Anti–SARS-CoV-2, and Architect Abbot SARS-CoV-2 IgG) in each of the three survey rounds. The seroprevalence is assumed to represent the actual proportion of people with prior SARS-CoV-2 infection, including asymptomatic cases not identified by official reports. The proportions of people with prior SARS-CoV-2 infection were 1.7 to 3.5 times higher than the prevalence of confirmed COVID-19 cases in Hiroshima Prefecture.

The cumulative numbers of cases of SARS-CoV-2 infection as officially reported by the Hiroshima Prefectural Government were 458 through the end of August 2020, 662 through the end of October 2020, and 4,831 through the end of January 2021. Based on these numbers, the prevalence of confirmed COVID-19 cases in Hiroshima Prefecture during these periods were estimated to be 0.02%, 0.02%, and 0.17%, respectively. Therefore, it was assumed that the actual numbers of people with prior SARS-CoV-2 infection, as determined by seroprevalence rates, were 1.7 to 3.5 times higher than the reported numbers in Hiroshima Prefecture (Fig. 3).

### Anti–SARS-CoV-2 antibody seroconversion rate

**Fig. 4:**
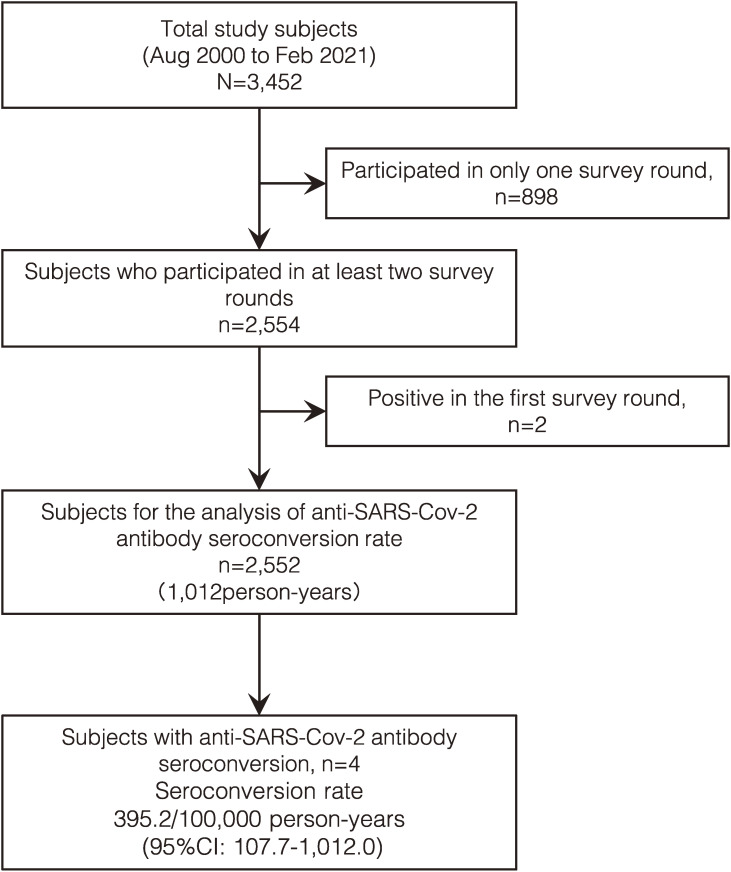
Anti-SARS-Cov-2 antibody seroconversion rate in Hiroshima Prefecture from August 2020 to February 2021 This figure shows the flow of the follow-up subjects on the study of anti-SARS-Cov-2 antibody seroconversion rate. From a total of 3,452 subjects who participated at least once in the three rounds of the study, 2,552 subjects were included in the analysis of anti-SARS-Cov-2 antibody seroconversion rate. Four subjects acquired anti-SARS-Cov-2 antibodies during the follow-up, indicating a seroconversion rate of 395.2 persons per 100,000 person-years.

The seroconversion rate was estimated using the person-years method in 2,552 participants who participated in at least two survey rounds and tested negative for anti–SARS-CoV-2 antibodies at the first round. Four participants seroconverted during 369,391 person-days (1,012 person-years) of observation, resulting in an estimated seroconversion rate of 395.2/100,000 person-years (95% CI, 107.7–1,012.0/100,000) between August 2020 and February 2021 (Fig. 4). None of the participants who participated in at least two rounds demonstrated seroreversion during the observation period.

### Comparison of seroprevalence with other prefectures

Table 3 compares the findings of the Japanese government report of confirmed COVID-19 cases in December 2020 with both the results of the third round of this study and those of the survey of seroprevalence of anti–SARS-CoV-2 antibodies conducted by the MHLW in a randomly sampled general population in Tokyo, Osaka, Miyagi, Aichi, and Fukuoka prefectures. The third round of our survey and the MHLW survey were conducted during almost the same period. In this comparison, a positive test was defined as positive results with two immunoassays, Elecsys Anti–SARS-CoV-2 (Roche Diagnostics International Ltd) and Architect Abbot SARS-CoV-2 IgG (Abbott Diagnostics). Therefore, one case in which the three reagents of Ortho anti-S Total, Ortho anti-S IgG and Abbott anti-N IgG were positive (case 6 in Supplementary Table 1), and one case in which the two reagents of Ortho anti-S Total and Ortho anti-S IgG were positive (case 7 in Supplementary Table 1) were excluded from the positive cases. The estimated prevalence of prior SARS-CoV-2 infection based on the seroprevalence of anti–SARS-CoV-2 antibodies in Tokyo, Osaka, Miyagi, Aichi, and Fukuoka prefectures were 2.9, 2.2, 2.5, 3.6, and 1.6 times higher than the prevalence of confirmed COVID-19 cases in these prefectures, respectively. In contrast, our study showed that the prevalence of prior SARS-CoV-2 infection based on the seroprevalence of anti–SARS-CoV-2 antibodies in Hiroshima Prefecture was only 1.2 times higher than the prevalence of confirmed SARS-CoV-2 infections.

**Table 3:** Comparison of anti-SARS-Cov-2 antibody seroprevalence and the prevalence of confirmed COVID-19 cases in five prefectures

**SARS-Cov-2 antibody seroprevalence**				**[B]** **Prevalence of COVID-19 based on Government Report of confirmed cases** **(%)**	**Ratio** **[A]/[B]**

**Study**	**Prefecture**	**Sample Size** **N**	**[A]** **Seroprevalence of ** **anti-SARS-CoV-2 antibodies (Roche & Abbott Positive) ** **N (%)**		
Results from the MHLW random sampling study (Dec 2020)	Tokyo	3,399	31 (0.91)	0.32*	2.9
	Osaka	2,746	16 (0.58)	0.26*	2.2
	Miyagi	2,860	14 (0.14)	0.06*	2.5
	Aichi	2,960	16 (0.54)	0.15*	3.6
	Fukuoka	3,078	6 (0.19)	0.12*	1.6

Results from this study (Jan-Feb 2021)	Hiroshima	2,351	5 (0.21)	0.17**	1.2

MHLW, Ministry of Health, Labour and Welfare of Japan

*Based on Japanese government report of confirmed COVID-19 cases on December 7, 2021

**Based on Japanese government report of confirmed COVID-19 cases on January 31, 2021

## Discussion

Monitoring the seroprevalence of anti–SARS-CoV-2 antibodies by region is essential to determine the percentage of people susceptible to SARS-CoV-2, as this percentage can be used to forecast future demand for medical care. As recommended by the WHO, an epidemiological survey on the seroprevalence of anti–SARS-CoV-2 antibodies is an effective tool to assess the scale of the outbreak, including asymptomatic cases [18, 22].

The seroprevalence rates in the first, second, and third rounds of the survey were 0.03%, 0.08%, and 0.3%, respectively. Although the seroprevalence rate gradually increased from the first to the third rounds, it remained very low. This indicates that in the 2020 fiscal year, before the introduction of vaccination in Hiroshima Prefecture, most people were susceptible to SARS-CoV-2. It is presumed that the Japanese were able to avoid infection because the mask wearing rate was extremely high and they were active in preventive actions [32].

Strengths of this study include the use of four different immunoassays to make the results robust, and the implementation of a large-scale, random sampling survey that was administered repeatedly in the same population to allow for longitudinal evaluation.

The longitudinal evaluation showed that the seroconversion rate between August 2020 and February 2021 was 395.2/100,000 person-years, and that no participants demonstrated seroreversion during this period. Although acquired antibodies wane with time, it has been reported that antibodies that have been acquired through infection are maintained for at least 6 to 10 months [33, 34]. The reason for the absence of seroreversion in this study may be that the survey was conducted less than a year after the first case of COVID-19 in Hiroshima Prefecture in May 2020.

The seroprevalence of anti–SARS-CoV-2 antibodies reported in population-based surveys in other countries has ranged from 1.09% to 17.1% [16, 21, 29, 35–40]. This variation may be attributed to differences between regions in the scale of the outbreak and the accuracy of antibody testing. It should be considered that false-positive results may occur in populations with a very low prevalence of infection when only a single antibody assay is used [16, 28–30]. Therefore, to obtain robust results, this study performed antibody testing using four different immunoassays and the evaluation was made with reference to the results of neutralizing antibody detection, changes over time in antibody measurement results, and subjective symptoms. We defined a positive result when at least two different tests were positive. The study of the Japanese Ministry of Health, Labor and Welfare was also conducted in a population with a very low prevalence and defined antibody-positive when both of the two reagents were positive [31].

Of the seven participants who tested positive, three (42.9%) were aware of their infection, while four (57.1%) were not. It has been shown that SARS-CoV-2 may be transmitted by asymptomatic carriers [10–12]. The percentage of asymptomatic SARS-CoV-2 infection cases has been reported to range from 16% to 33.3% [13–15]. Vaccination is important to break the chain of infection, as is determining close contacts of the infected person, conducting epidemiological surveys to identify infected people using polymerase chain reaction (PCR) testing, and screening for asymptomatic patients by providing people with free PCR testing. The gap between the reported number of cases and the estimated number based on seroprevalence depends on the presence of measures to detect asymptomatic patients as well as on the surveillance system available in the country or region. In Hiroshima Prefecture, active measures have been taken, including the establishment of PCR centers and the implementation of periodic PCR testing in medical and nursing care facilities. The gap between antibody seroprevalence and the results of the government report on confirmed COVID-19 was smaller in Hiroshima than in other prefectures. This finding suggests that the detection rate of asymptomatic cases in Hiroshima Prefecture was higher than that in other prefectures.

### Limitations

There were several limitations to our study. First, even though the survey targeted a randomly sampled general population, the fact that participation was voluntary may have resulted in selection bias, with a higher likelihood of participation among people who suspected that they were infected with SARS-CoV-2. However, the percentage of participants who reported not being infected with SARS-CoV-2 before or during the survey was 95% to 97%. In addition, 2,554 of 3,452 participants responded to the survey at least twice, indicating that a large number participated regardless of awareness of their past infection. Second, the participation rate was lower than expected. Therefore, the absolute accuracy of 0.15% could not be guaranteed. However, a longitudinal study on the seroprevalence of SARS-CoV-2 antibody in a randomly selected sample from the general population is rare and novel in Japan. Third, antibody seropositivity is not detected in the very early phase of SARS-CoV-2 infection [41–43], and thus it is possible that newly infected cases could not be identified. However, antibody testing was performed three times in the same population, making it likely that participants who tested more than once would be positive if they were infected. Finally, some participants with prior infection may not have been detected because of the natural waning of antibodies, although this is unlikely because the survey was conducted less than a year after the outbreak of SARS-CoV-2 infection.

## Conclusions

A longitudinal study on the seroprevalence of anti–SARS-CoV-2 antibodies in a randomly sampled general population was conducted in Hiroshima Prefecture. Based on the number of participants who tested positive by at least two of the four immunoassays, the seroprevalence of prior SARS-CoV-2 infection was determined. The survey revealed that the number of people with anti–SARS-CoV-2 antibodies in Hiroshima Prefecture increased tenfold in a half year, although most of these individuals were antibody negative in 2020, prior to the introduction of SARS-CoV-2 vaccines. The difference between antibody seroprevalence and the prevalence of confirmed COVID-19 cases officially reported in Hiroshima was smaller than that in the other prefectures, suggesting that asymptomatic patients were more actively detected in Hiroshima. Since antibody titers have been shown to decline over time [44–49], monitoring by antibody testing remains important as a specific measure to control COVID-19. Monitoring antibody seroprevalence is useful from a public health perspective to assess herd immunity for diseases with asymptomatic infections such as COVID-19.

## Abbreviations

SARS-CoV-2: Severe acute respiratory syndrome coronavirus 2
WHO: World Health Organization
COVID-19: Coronavirus disease 19
IgG: Immunoglobulin G
MHLW: Ministry of Health, Labour, and Welfare
PCR: Polymerase chain reaction

## Supplementary information

**Additional file 1:** **Supplementary Table 1.** Detailed information on 39 cases positive for anti-SARS-CoV-2 antibody with one or more reagents.

## Declarations

### Availability of data and materials

The datasets used and analyzed during the current study are available from the corresponding author on reasonable request.
